# Differential Cortical Gray Matter Deficits in Adolescent- and Adult-Onset First-Episode Treatment-Naïve Patients with Schizophrenia

**DOI:** 10.1038/s41598-017-10688-1

**Published:** 2017-08-31

**Authors:** Chengcheng Zhang, Qiang Wang, Peiyan Ni, Wei Deng, Yinfei Li, Liansheng Zhao, Xiaohong Ma, Yingcheng Wang, Hua Yu, Xiaojing Li, Pingping Zhang, Yajing Meng, Sugai Liang, Mingli Li, Tao Li

**Affiliations:** 1 Mental Health Center and Psychiatric Laboratory, the State Key Laboratory of Biotherapy, West China Hospital of Sichuan University, Chengdu, Sichuan 610041 China; 2 West China Brain Research Center, West China Hospital of Sichuan University, Chengdu, Sichuan 610041 China

## Abstract

The current study aimed to explore age-variant trait differences of cortical gray matter volume (GMV) in a unique sample of first-episode and treatment-naïve patients with schizophrenia. A total of 158 subjects, including 26 adolescent-onset patients and 49 adult-onset patients as well as 83 age- and gender-matched controls were scanned using a 3T MRI scanner. Voxel-based morphometry (VBM) following Diffeomorphic Anatomical Registration Through Exponentiated Lie algebra (DARTEL) was used to explore group differences between patients and controls in regional GMV. We found that patients with schizophrenia had decreased GMV in the left parietal postcentral region that extended to the left frontal regions, the right middle temporal gyrus, the occipital lobe and the right cerebellum posterior pyramis. Further analysis showed a distinct pattern of gray matter alterations in adolescent-onset patients compared with both healthy controls and adult-onset patients. Relative to healthy controls, adolescent-onset patients showed GMV alterations in the left parietal postcentral gyrus, parahippocampal gyrus and right cerebellum posterior pyramis, while GMV deficits in adult-onset patients were focused on the cingulo-fronto-temporal module and right occipital regions. Our study identified differential cortical gray matter deficits between adolescent- and adulthood-onset patients with schizophrenia, which suggests that the cortical abnormalities in schizophrenia are likely adjusted by the developmental community structure of the human brain.

## Introduction

Previous studies have shown cerebral cortical gray matter abnormalities in patients with schizophrenia^1–3^. However, the results are inconsistent and make it difficult to reconcile the notion that different cerebral regions may be linked with the disorder in different studies. While Matsumoto *et al*. observed that patients with schizophrenia have reduced gray matter volume (GMV) in temporal lobe regions^4^, others have reported increased GMV or no structural differences at all in schizophrenic patients^5, 6^. Possible explanations for the inconsistent findings are the varying of neuroimaging methodologies adopted in studies and the heterogeneity of the disorder itself^7^. Furthermore, it is not clear if and how these abnormalities are adjusted by the brain’s developmental process.

Studying adolescent-onset patients with schizophrenia offers a unique approach to understanding the pathophysiological process of the disorder through exploring the interplaying outcomes of disease-associated pathology and age-related brain development^8^. However, the majority of imaging studies in schizophrenia have been confined to adult subjects due to the availability of the sample^9^. In contrast, there have been few studies that have explored whole-brain changes in early-onset patients with schizophrenia on a voxel-by-voxel basis^10^. Moreover, the structural cerebral changes revealed in early-onset schizophrenia have previously shown great inconsistencies. For example, Matsumoto *et al*. reported that the total volume and GMV of the right superior temporal gyrus was significantly lower in patients with schizophrenia than in healthy controls^4^. Taylor *et al*., however, found increased volume in the temporal lobe regions of childhood-onset patients with schizophrenia (COS)^11^.

Compared with the developmental course of white matter, cortical gray matter follows an inverted U-shape trajectory with greater regional variation^12, 13^. Studies have also found that the synaptic density is increased above adult levels during early postnatal brain development^13^. In addition, the synapses go through a process with brain regions responsible for primary motor and sensory functions maturing earlier than those serving higher-order functions^14, 15^. Thompson *et al*. reported that there are parietal and motor cortex deficits at the earlier stage of schizophrenia at baseline, while the deficits of prefrontal, supplementary motor and temporal deficits appeared or increased over the course of the illness^16^. Alexander-Bloch *et al*. found that patients with COS showed a converged brain development trajectory with controls in the parietal regions while remaining divergent in the anterior (frontal and temporal) regions, with a pattern similar to that seen in adults with schizophrenia. This may indicate that patients with adult-onset schizophrenia have undergone a similar process of parietal-frontal wave deficits prior to the onset of illness, while patients with child-onset schizophrenia may have an inflation of the progressive pattern of normal posterior-anterior development^17^.

Age-related changes in gray matter throughout normal adolescence might be dynamic, with substantial thinning of cortical gray matter starting initially in primary areas and occurring later in the secondary cortices of the frontal and parietal lobes and finally in the temporal lobes^18^. In early-onset schizophrenia, investigations using magnetic resonance imaging (MRI) have suggested that during childhood, the temporolimbic and cingulate brain regions do not show volumetric changes^19^. Greenstein *et al*. performed a study with repeated MRI scans in 70 patients with COS aged 7–26 years old and reported a reduction of overall mean cortical thickness, especially in the prefrontal and temporal regions^20^. However, other researchers have found that early-onset patients with schizophrenia have parietal brain region deficits extending anteriorly into the temporal lobes and reaching the cingulate gyrus^16^. The difference between adult- and early-onset schizophrenia, in terms of the relationship between progressive changes and clinical correlates, may occur as a result of different brain development stages at the time of the first episode. In addition, a few studies have found that individuals with adult-onset schizophrenia have more widespread white matter deficits in the parietal lobe compared with adolescent-onset patients^21^.

It should be noted that most longitudinal studies of first-episode psychosis comprise both adolescents and adults, making it difficult to determine the influence of normal development, and the effects of antipsychotic treatment on the results prior to scanning. Therefore, to avoid these confounding factors and minimize the impact of illness duration and medication on patients, our current study focused on a unique sample of schizophrenic patients who were experiencing their first episode and were treatment-naïve prior to scanning. We adopted an individualized Diffeomorphic Anatomical Registration Through Exponentiated Lie algebra (DARTEL)-based template to provide more precise spatial alignment^22^, with the aim of assessing the effect of age at the onset of schizophrenia on gray matter abnormalities and identifying the gray matter difference between adolescent- and adult-onset patients.

## Results

### Demographic characteristics and clinical information

The demographic characteristics and clinical information of the participants are shown in Table 1. The patients with schizophrenia were aged between 14 and 45 (mean age = 23.71, SD = 9.09) years, their duration of education ranged from 7 to 19 (mean duration = 11.64, SD = 3.54) years. Healthy controls aged from 16 to 45 (mean age = 27.84, SD = 9.12) years, their duration of education was 8 to 19 (mean duration = 13.44, SD = 3.62) years. There were no statistically significant differences in the distribution of sex (χ^2^ = 0.11, P = 0.75), age (t = −1.42, P = 0.16) or education years (t = −0.37, P = 0.71) between patients and controls.

**Table 1:** Demographic variables and clinical characteristics of adolescent-onset and adult-onset antipsychotic-naïve, first-episode schizophrenia patients and healthy control subjects.

Characteristic	Adolescent participants			P	Adult participants			P
	FES group	Healthy group	comparison		FES group	Healthy group	comparison	
	(N = 26)	(N = 26)	t/*χ* ^2^		(N = 49)	(N = 57)	t/*χ* ^2^	
Age (years); mean (SD)	16.87(1.05)	16.81(0.75)	0.12	0.22	32.88(5.94)	32.88(6.26)	0.03	0.86
Education (years); mean (SD)	10.35(1.38)	11.19(1.02)	5.81	0.02	12.33(4.12)	13.02(4.21)	0.01	0.87
Sex (male: female)	13:13	13:13	0	1	20:29	26:31	0.03	0.62
TMV; mean (SD)	1,254.50 (137.15)	1,262.87 (85.69)	−0.26	0.79	1,166.91 (105.73)	1,214.48 (109.84)	−2.26	0.03
Illness duration (months); mean (SD)	3.61(3.50)				4.85(7.29)			
Age onset (years); mean (SD)	16.51(1.01)				32.48(5.83)			
Global assessment function; mean (SD)	27.96(8.19)				27.31(8.17)			
**PANSS scores; mean (SD)**								
Total	93.42(16.43)				89.22(17.15)			
Negative	20.46(8.19)				16.7(6.38)			
Positive	25.12(4.92)				25.89(5.85)			
General	47.85(9.88)				46.63(9.89)			
Thought disturbance	13.65(3.74)				14.11(3.59)			
Activation	9.12(2.86)				9.43(3.77)			
Paranoid	10.62(2.00)				11.04(2.92)			
Impulsive aggression	16.85(5.14)				16.28(4.67)			
Anergia	9.77(4.88)				7.87(3.46)			
Depression	9.15(3.94)				8.74(4.16)			

Notes: PANSS, Positive and Negative Syndrome Scale; FES, antipsychotic-naive first-episode schizophrenia; TMV, total gray and white matter volume.

Compared with their matched controls, there were no significant differences in sex or age in the adolescent- and adult-onset groups, except fewer education years in the adolescent-onset group (t = 5.81, P = 0.02) and smaller total gray and white matter volumes in the adult-onset group (t = −2.26, P = 0.03). There were no significant differences in PANSS, GAF and DUP scores between adolescent- and adult-onset patients with schizophrenia (Table 1).

### GMV alterations in adolescent- and adult-onset patients with schizophrenia

**Figure 1:**
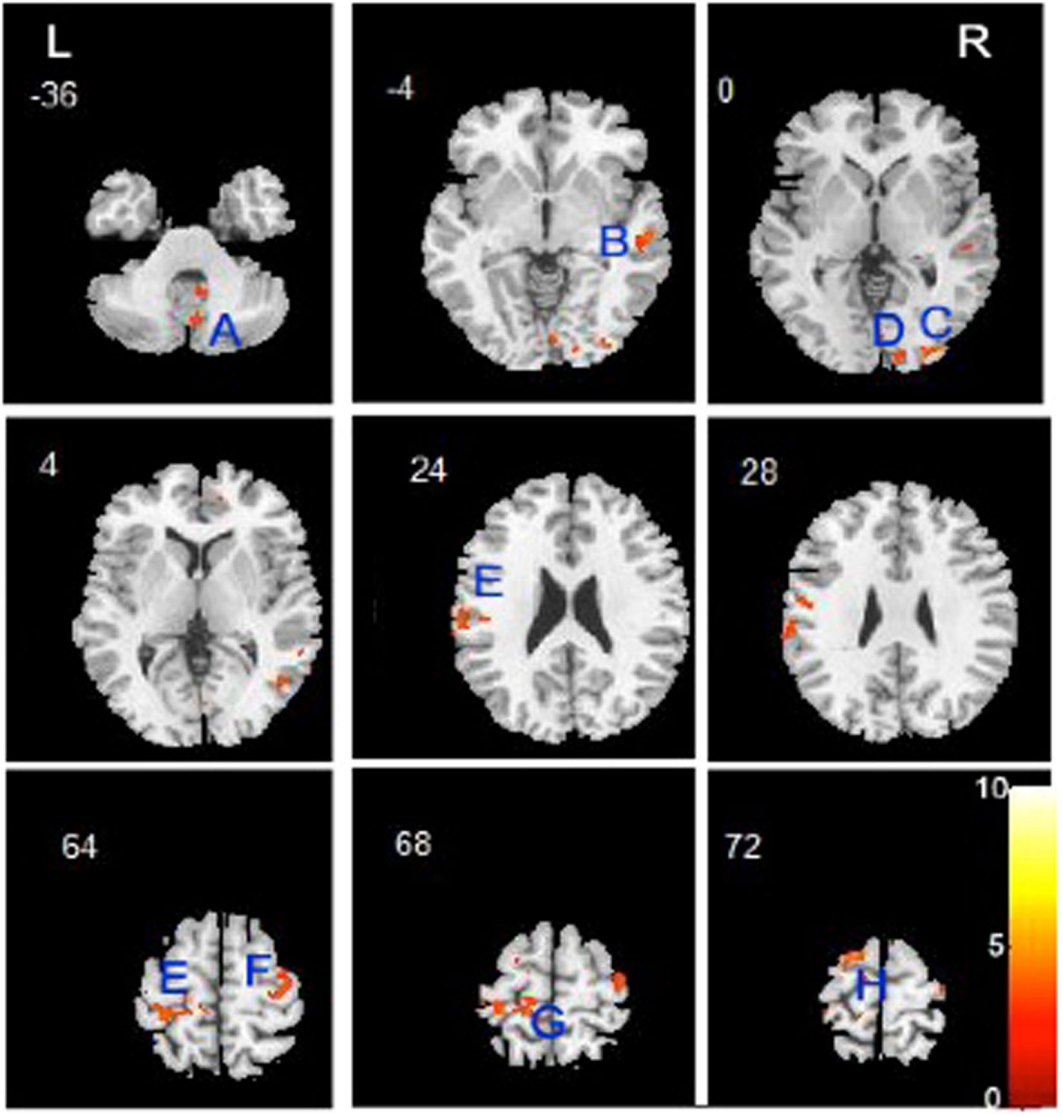
Relative to healthy comparison subjects, patients with drug-naïve, first-episode schizophrenia showed significantly reduced gray matter volume. (**A**) Right cerebellum posterior pyramis; (**B**) Right middle temporal gyrus; (**C**) Right middle occipital gyrus; (**D**) Right occipital cuneus gyrus; (**E**) Left parietal postcentral gyrus; (**F**). Right frontal precentral gyrus (**G**) Left frontal precentral gyrus; (**H**) Left superior frontal gyrus.

We found that patients with schizophrenia had decreased GMV in the left parietal postcentral region extending to the left frontal regions, right middle temporal gyrus, occipital region and right cerebellum posterior pyramis (Fig. 1 and Table 2).

**Table 2:** Gray matter volume comparison between drug-naïve, first-episode schizophrenia patients and healthy controls.

Comparison	Regions	L/R	X	Y	Z	Cluster size	T value
Patients < health subjects	Parietal postcentral gyrus	L	−10	−38	69	580	4.81
	Frontal precentral gyrus	L	−13	−35	70	608	4.42
	Superior frontal gyrus	L	−14	1	73	315	4.71
	Frontal precentral gyrus	R	38	−11	66	516	4.03
	Middle temporal gyrus	R	54	−29	−5	198	4.53
	Middle occipital gyrus	R	31	−91	−1	156	4.26
	Occipital cuneus gyrus	R	14	−95	0	157	3.76
	Cerebellum posterior pyramis	R	4	−71	−31	159	3.77

**Figure 2:**
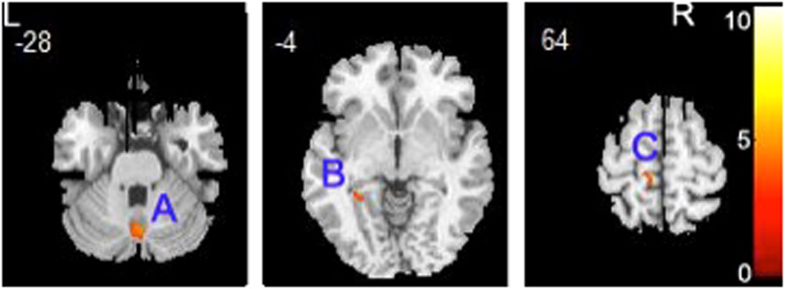
Relative to healthy controls, patients with adolescent-onset schizophrenia showed gray matter volume deficits in the left parietal postcentral gyrus, parahippocampal gyrus and right cerebellum posterior pyramis (**A**). Right cerebellum posterior pyramis (**B**). Left parahippocampal gyrus (**C**). Left parietal postcentral gyrus.

Patients with adolescent-onset schizophrenia showed GMV reductions in three discrete clusters (Fig. 2 and Table 3), corresponding to the left parietal postcentral gyrus, the parahippocampal gyrus, and the right cerebellum posterior pyramis (Fig. 2 and Table 3). There was no increased GMV in any brain region in the adolescent-onset group.

**Table 3:** Decreased gray matter volume in patients with adolescent-onset, drug-naive first-episode schizophrenia.

Comparison	Regions	L/R	X	Y	Z	Cluster size	T value
Patients < healthy subjects	Parietal postcentral gyrus	L	−10	−38	70	69	4.22
	Parahippocampal gyrus	L	−31	−44	−7	100	3.35
	Cerebellum posterior pyramis	R	4	−70	−29	200	5.27

**Figure 3:**
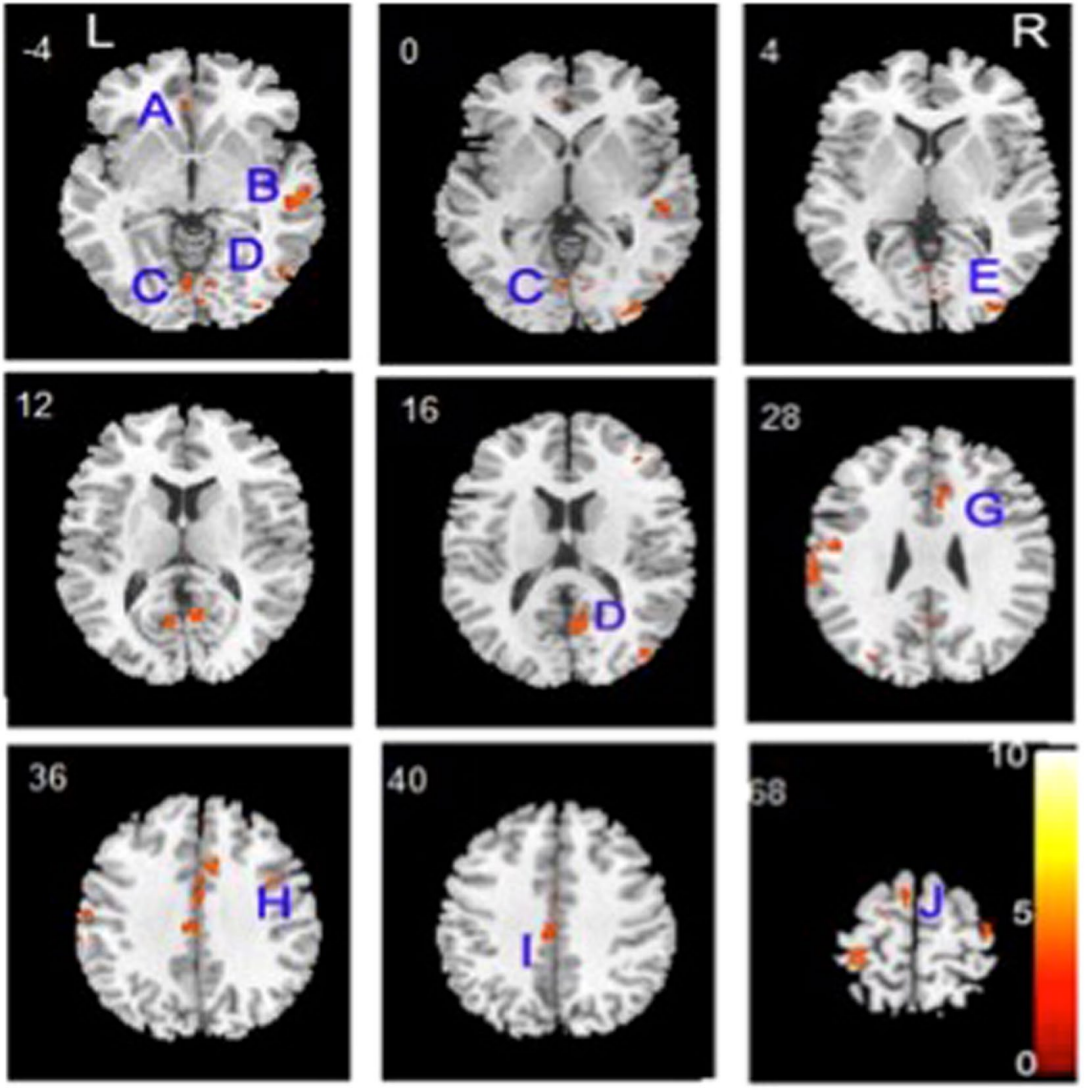
Relative to healthy comparison subjects, the gray matter volume loss of adult-onset, drug-naïve first-episode schizophrenia patients was centered on the cingulo-fronto-temporal module and right occipital regions. (**A**) Left anterior cingulate; (**B**) Right middle temporal gyrus; (**C**) Left occipital lobe lingual gyrus; (**D**) Right posterior cingulate; (**E**) Right middle occipital gyrus; (**G**) Right cingulate gyrus; (**H**) Right superior frontal gyrus; (**I**) Left cingulate gyrus; (**J**) Left superior frontal gyrus.

In the adult-onset group, patients showed GMV reductions in the right temporal-occipital regions and bilateral cingulate region relative to the matched controls (Fig. 3 and Table 4), which likely represented the right superior frontal gyrus and extending to the right middle temporal gyrus and middle occipital gyrus. There were also no increases in GMV in any brain regions in the adult-onset group compared with controls.

**Table 4:** Decreased gray matter volume in patients with adult-onset, drug-naive first-episode schizophrenia.

Comparison	Regions	L/R	X	Y	Z	Cluster size	T value
Patients < healthy subjects	Superior frontal gyrus	L	−15	−3	71	168	3.67
	Frontal precentral gyrus	L	−13	−35	69	112	4.08
	Occipital lobe lingual gyrus	L	−2	−80	−2	103	4.25
	Anterior cingulate	L	−3	33	−7	225	3.41
	Cingulate gyrus	L	−2	−24	38	320	3.95
	Superior frontal gyrus	R	24	49	24	151	3.98
	Middle temporal gyrus	R	63	−18	−5	168	4.71
	Middle occipital gyrus	R	34	−90	3	579	4.50
	Posterior cingulate	R	8	−56	13	730	4.10
	Cingulate gyrus	R	7	16	35	889	4.68

### Correlations between GMV and clinical assessments

We found a significant negative correlation between the GMV of the left parahippocampal gyrus and the paranoid subscale scores of the PANSS in the adolescent-onset patients (r = −0.47, P = 0.02, uncorrected). In the adult-onset patients, the GMV of the right posterior cingulate was positively correlated with the paranoid subscale (r = 0.32, P = 0.04, uncorrected) and the activation subscale (r = 0.32, P = 0.03, uncorrected). There was also a significant positive correlation between the GMV of the left superior frontal gyrus and depression subscale scores (r = 0.33, P = 0.03, uncorrected). However, above significant correlations were not maintained after Bonferroni correction for multiple tests.

## Discussion

In the present study, by studying a unique sample of first-episode and treatment-naïve patients with schizophrenia, we found that patients with schizophrenia had decreased GMV in the left parietal postcentral region extending to the left frontal regions, right middle temporal gyrus, occipital region and right cerebellum posterior pyramis. Most importantly, with a voxel-based morphometry analysis approach, we demonstrated for the first time a distinct pattern of gray matter alterations in adolescent-onset patients compared with both healthy controls and adult-onset patients. Relative to healthy controls, adolescent-onset patients showed GMV alterations in the left parietal postcentral gyrus, parahippocampal gyrus and right cerebellum posterior pyramis, while GMV deficits in adult-onset patients focused on the cingulo-fronto-temporal module and the right occipital regions.

Significant changes in cortical gray matter have been demonstrated in a large number of studies on schizophrenia and confirmed by meta-analysis^3^. Our findings indicated reduced GMV in the parietal, frontal, temporal and occipital cortices in first-episode and treatment-naïve patients with schizophrenia, which is consistent with previous studies, including our own^23^. These results implicate a wide range of brain changes within the cortices during the earlier stages of the illness and without the effects of antipsychotic medication^24^.

We were particularly motivated by the issue that neurodevelopmental processes continue throughout late adolescence and that an episode of the illness may affect the pattern of GMV alteration in schizophrenic patients, and therefore, we explored the effects of age of onset on GMV in adolescent- and adult-onset subgroups of patients. Using voxel-based analysis, we revealed a regional reduction of GMV in the left parietal postcentral gyrus and parahippocampal gyrus in adolescent-onset patients. This result is consistent with some previous studies in patients with early-onset schizophrenia^21^. However, because regional alterations of GMV have been frequently observed in various brain regions of early-onset patients rather than being limited to the above brain regions alone^25, 26^, and because abnormal structural changes of gray matter are deemed to be a fundamental part of the pathophysiology of schizophrenia, the observation of gray matter abnormalities in cortical regions (i.e., the parietal postcentral and parahippocampal regions) in individuals with adolescent-onset schizophrenia in the present study may indicate the brain areas in which the disorder originates. However, our studies were not completely consistent with findings from some previous studies on early-onset schizophrenia^27^, which have shown that frontal gray matter loss may be present in children and adolescents. The difference between studies may be due to the samples included. Our study included adolescent-onset patients only, while others have grouped childhood- and adolescent-onset schizophrenia patients together. Although both childhood-onset and adolescent-onset patients are usually defined as early-onset schizophrenia, it is likely that they have significant differences in precursors, disease course and neurodevelopment.

The cerebellum has been suggested to play an important role in the pathophysiology of schizophrenia^28, 29^. In the present study, we also found reduced GMV in the right cerebellum posterior pyramis in adolescent-onset patients, which is in line with previous findings from earlier studies^30^. The vermis has a connection with the thalamus and limbic system via the fastigial nucleus^31^, and abnormality in the cerebellum vermis may disrupt the function of the thalamus-limbic-frontal circuit in patients with schizophrenia^32^. Our results further confirm the existence of structural abnormality of the cerebellum posterior pyramis in the early stages of the disease.

Consistent with previous studies^17^, in the current study, we found the adult-onset patients with schizophrenia showed more widespread GMV reductions than did adolescent-onset patients. This reduced GMV was noted in the cingulo-fronto-temporal module and occipital regions in addition to parietal regions in adult-onset patients with schizophrenia. In addition, a study by Kyriakopoulos *et al*. found that white matter deficits presented predominantly in the frontal and temporal regions in adult-onset patients with schizophrenia and less in the parietal lobe of adolescent-onset patients, suggesting that the abnormal white matter in the frontal regions in schizophrenia may be related to the developmental stage at the time of illness onset^21^. Our findings indicate a similar pattern to that found by Kyriakopoulos *et al*. with respect to gray matter abnormalities. Anatomically, it has been shown that the anterior cingulate cortex has strong reciprocal connections with the superior temporal cortex^33^. This result fits with the proposal that young adult patients with schizophrenia show significant cortical thinning within the occipital, cingulate and frontopolar cortices^21, 34^, and it is also consistent with evidence that age-constant deficits in cortical thickness overlap significantly with the cingulo-fronto-temporal module in schizophrenia^17^. Interestingly, a previous study showed that the initial and severe reduction in the cortical thickness of frontal and temporal lobe regions in COS patients may partially be “normalized” in early adulthood^21^. Furthermore, reduced cortical thickness has been reported in repeated scans from 70 patients aged 7–26 years and diagnosed with COS^20, 35^. In the normal brain, partial GMV peaks at approximately 11 years old, whereas GMV in the temporal occipital module peaks at approximately age 16.7 years; there is then a progressive reduction in GMV after the peak^17^. Studies have also implicated the normal maturational process of synaptic/dendritic pruning during the important time of adolescence, which may offer insight into understanding the neurobiological mechanisms underlying “normalized” changes in adolescence and/or more widespread reductions of GMV in adult-onset patients. Thus, it should be strongly suggested to consider the age of onset of the illness as a significant factor when examining progressive brain changes in schizophrenia.

The present study has several advantages. First, we recruited a group of first-episode, treatment-naïve patients with schizophrenia in our analysis to minimize confounding factors due to medication use and the chronicity of the illness. Previous studies have indicated that the cortical volume abnormalities in patients with schizophrenia might have genetic or shared environmental origins rather than being a consequence of drug treatments^36^; however, most previous studies recruited patients with schizophrenia who were being treated with antipsychotics at the time of the scan and therefore could not completely eliminate the effects of antipsychotic treatment^37–39^. Second, compared with the classic ROI method, previous studies employing ROIs with pre-specification have done so on a relatively small number of hypothesized brain regions in patients with schizophrenia^40^. In fact, voxel-based morphometry has proven to be somewhat more sensitive than ROI methods in terms of attempting to systematically review a large body of neuroanatomic literature^34^.

The results of this study should be interpreted in the context of the following limitations. Schizophrenic patients and control subjects showed significant differences in terms of educational years. However, we found there was no association between educational years and abnormal GMV, so this difference may not alter the overall significance of our results^41^. Furthermore, a longitudinal rather than cross-sectional design is required to verify the dynamic changes of GMV in adolescent- and adult-onset patients.

In conclusion, our research found that reduced GMV in adolescent-onset patients with schizophrenia mainly focused on the parietal cortical regions, while adult-onset patients showed reduced GMV in the cingulo-fronto-temporal module and right occipital regions. The differential cortical gray matter deficits in adolescent- and adulthood-onset patients with schizophrenia may suggest that the cortical abnormalities in schizophrenia are likely adjusted by the developmental community structure of the human brain.

## Materials and Methods

### Subjects and clinical assessments

A total of 158 subjects participated in this study, including 75 patients with first-episode schizophrenia and 83 healthy controls. All of the patients were recruited from inpatient and outpatient psychiatric units at the Mental Health Centre of the West China Hospital from 2008–2013. The patients were assessed by trained psychiatrists using the Structured Clinical Interview for DSM-IV Patient version (SCID-P) after their first presentation to mental health services. Diagnoses were assigned according to the diagnostic criteria for schizophrenia as specified in the DSM-IV. To be included, an individual was required to fulfill the following inclusion criteria for the present study: age between 14 and 45 years, Han Chinese, right-handed, intelligence quotient ≥70, experiencing their first episode of schizophrenia, and treatment-naïve or no more than 3 days of antipsychotic treatment before clinical assessment and MRI scan. Information on previous treatment, the duration of untreated psychosis (DUP) and the Global Assessment of Functioning (GAF) score were collected at diagnosis^42^. All of the patients underwent further evaluation of their clinical symptoms using the Positive and Negative Syndrome Scale (PANSS), which provided a total score and syndrome scores for the following categories: Negative, Positive, General, Thought disturbance, Activation, Paranoid, Impulsive aggression, Anergia, and Depression^43^. All of the items were rated from 1 (absent) to 7 (extreme) according to standardized instructions.

Healthy participants were recruited between 2008 and 2013 via word-of-mouth or posting advertisements. The inclusion criteria for controls were as follows: age between 16 and 45 years, Han Chinese, right-handed, and intelligence quotient ≥70. All of the controls were screened for a lifetime absence of Axis I illness or DSM-IV psychiatric illnesses with the SCID Non-Patient version (SCID-NP). In addition, healthy participants were interviewed to ascertain that there was no psychiatric illness in their first-degree relatives. Participants with a history of any major psychiatric disorders (such as affective and schizoaffective disorders), intellectual disability, head trauma, any substance abuse (except tobacco), alcohol abuse disorder, any major nervous system diseases, and severe physical diseases were excluded. All subjects were Han Chinese and right-handed as assessed by the Annett Handedness Scale^44^.

Patients were assigned into 2 subgroups: patients with adolescent-onset (aged 14 to 17 years) or with adult-onset (aged 18 to 45 years) schizophrenia^21^. Two subgroups of healthy controls were age- and sex-matched with the adolescent-onset and adult-onset patients. Written informed consent was obtained from all participants or their guardians if they were under 18 years of age. All methods were performed in accordance with the relevant guidelines and regulations approved by the Institutional Review Board of West China Hospital of Sichuan University.

### MRI data acquisition

All scanning was performed on a 3T MRI system (EXCITE; General Electric, Milwaukee, Wisconsin) using an eight-channel phased-array head coil without upgrading of the scanner software or hardware except daily management and maintenance during 2008 and 2013 in order to keep data consistence and high quality^45–47^. Foam padding and earplugs were used to minimize head movement and scanner noise. High-resolution T1 images were obtained by the 3D spoiled gradient echo sequence (SPGR) from all participants. The protocols used were as follows: TR = 8.5 ms, TE = 3.93 ms, flip angle = 12°, thickness of slice = 1 mm, single shot, field of view (FOV) = 24 × 24 cm^2^, matrix = 256 × 256, size of voxel = 0.47 × 0.47 ×1 mm^3^. In total, 156 slices of axial images were collected from each brain. The raw MRI data were checked by two experienced neuroradiologists. No artifacts or gross anatomical abnormalities were observed for any subject^48^.

### Image preprocessing

Image files in DICOM format were transformed to NIfTI format using dcm2nii software (http://www.cabiatl.com/mricro/mricron/dcm2nii.html). The 3D T1-weighted images were processed using voxel-based morphometry in Statistical Parametric 8 (SPM8) software (http://www.fil.ion.ucl.ac.uk/spm) and ran on the MATLAB 7.3 platform (MathWorks, Inc., Natick, MA, USA). Next, using the Medical Image NetCDF (MINC) software package (http://wiki.bic.mni.mcgill.ca/index.php/MINC), we adopted the non-parametric non-uniformity intensity normalization technique (N3) to strengthen the non-uniformity of the high-magnetic field signals obtained in the transformed images. Because we wanted to study voxel-wise changes between schizophrenic patients and control subjects across the whole brain, the use of non-linear deformations to register native scans into a common space played an important role in carrying out the process with greater accuracy. Thus, voxel-based analyses using different practical methodologies for the automated segmentation and registration of the brains through a new segment tool implanted in SPM were carried out for the investigation of gray matter morphometry. The DARTEL algorithm, which is an automated, unbiased, and nonlinear template building program, was adopted using a similar strategy to that described by Asami *et al*.^7^. The specific procedure was performed as follows: first, gray matter was automatically segmented using tissue-signal intensity values or tissue priors for the distribution of brain tissue type (such as gray matter, white mater and cerebrospinal fluid); second, the gray matter tissue class images were spatially normalized onto the subject-specific template space, and the signal intensity of the normalized maps was modulated using the determinant of the Jacobian of the transformation; third, the population gray matter template was created by the DARTEL algorithm using all of the subject-specific templates of the gray matter tissue images; fourth, the normalized gray matter tissue maps for each subject were spatially and nonlinearly normalized to the population template and then modulated; fifth, the population GM template was aligned to the stereotaxic space using a 12-parameter affine transformation, and all of the individual tissue class images that were residing in the population template space were then co-registered to the stereotaxic atlas space using the same affine transformation; and finally, the gray matter tissue class images were smoothed with a 4-mm FWHM Gaussian kernel. A relatively small Gaussian kernel size of 4 mm was chosen to maintain the sensitivity to detect small brain structural changes in patients and, more importantly, to improve the signal-to-noise ratio. The volumes of gray matter and white matter were estimated from the smoothed images using custom MATLAB code (http://www.cs.ucl.ac.uk/staff/G.Ridgway/vbm/get_totals.m)^49^.

### Statistical analyses

Two-sample t-tests were performed to assess the differences in age, years of education and clinical data between the patient and control groups. For the sex data, χ^2^ tests were applied. The two-sample t-test or χ^2^ tests were used to compare the distributions and differences of continuous and categorical data between subgroups as appropriate.

The smoothed images, which were generated from image preprocessing, were entered into two-step analysis. Voxel-wise comparisons of GMV were performed using two-sample t-tests between patients and controls. Because age, sex, and total brain volume are potential confounding factors affecting regional cerebral volumes, their effects were removed by controlling the covariance. The above analyses were then performed in the two onset age-groups (age- and sex-matched) to examine the different patterns between adolescent- and adult-onset patients. Explicit masking, which was constructed from the overall 158 subjects using the “SPM Masking Toolbox” (http://www0.cs.ucl.ac.uk/staff/g.ridgway/masking/), was applied to ensure that only voxels within the gray matter mask were analyzed. We set the significant differences at the threshold of P < 0.05 at a FWE corrected cluster level and voxel-wise t > 3.12^50^. Each individual cluster that showed significant differences between groups was defined as a region of interest (ROI). The GMVs of individual ROIs were extracted from each subject and compared between groups using ANOVAs in SPSS 16.0. To assess the functional significance of GMV alterations in patients, the values of GMV in each ROI were correlated with the PANSS and the DUP scores. The correlation analysis was carried out using Pearson’s correlation or Spearman’s correlation if the variables showed non-normal distribution in SPSS, in all patients (including the adolescent and adult patients) and in the adolescent and adult groups respectively. The significant correlation was set at P < 0.05 (two-tailed) after Bonferroni multiple comparisons.
